# Supplementary material for: The Influence of Capping Layers on Tunneling Magnetoresistance and Microstructure in CoFeB/MgO/CoFeB Magnetic Tunnel Junctions upon Annealing
Source: Nanomaterials (Basel). 2023 Sep 19;13(18):2591. doi: 10.3390/nano13182591 (PMC10534786; doi:10.3390/nano13182591)
Supplement: Supplementary file 1 [file nanomaterials-13-02591-s001.zip › nanomaterials-2538194-supplementary.pdf]

Supplementary Information

The Influence of Capping Layers on Tunneling Magnetoresistance and Microstructure in CoFeB/MgO/CoFeB Magnetic Tunnel Junctions upon Annealing

Geunwoo Kim [1], Soogil Lee [1,2,*], Sanghwa Lee [1], Byonggwon Song [3], Byung-Kyu Lee [3], Duhyun Lee [3], Jin Seo Lee [4], Min Hyeok Lee [5], Young Keun Kim [5] and Byong-Guk Park [1,*]

Table of content

- **Supplementary Information S1. The diffusion of the W capping layer**
- **Supplementary Information S2. The diffusion of B atoms and crystallization of the CoFeB layers**

Supplementary Information S1. The diffusion of the W capping layer

To compare the degree of diffusion between capping layer materials, we performed X-ray photoemission spectroscopy (XPS) depth profile experiments. Figures S1(a), S1(b), and S1(c) present the composition depth profiles of MTJs as a function of the etching time for Pt-, Ta-, and W-capped MTJs, respectively. The step of etching time is 10 s. The film structures used in XPS experiments are the same as the MTJ devices described in the main text. Based on the obtained XPS spectra, we first determined the relative positions of each layer within MTJ films at which the XPS spectra of each layer's element reach their peak intensities. For example, for the Pt-capped MTJ, we determined that the XPS spectra acquired after 40 s of etching represent the chemical states of the top CoFeB layer because the Co 3*d* and Fe 3*d* peaks reach their maximum intensities at this point. The dashed line and grey area on each graph represent the assumed centers of the capping and top CoFeB layers, respectively.

Figure S1(d) presents the XPS spectra of Pt 4*f* peaks measured at the centers of the Pt capping and top CoFeB layers for the Pt-capped MTJ. Notably, the intensity of the Pt 4*f* peaks measured in the top CoFeB layer is comparable to that measured in the Pt capping layer. This indicates significant diffusion of Pt atoms into the top CoFeB layer, which is consistent with the observations of transmission electron microscopy (TEM) measurements [Figure 2 in the main text]. In contrast, for the Ta-capped MTJs [Figure S1(e)], the intensity of Ta 4*f* peaks measured in the top CoFeB layer is noticeably decreased compared to those measured in the Ta capping layers. This result indicates that the annealing-induced diffusion of Ta atoms into the top CoFeB layer is not significant, which is supported by the TEM results [Figure 3 in the main text]. Figure S1(f) shows the XPS spectra of W 4*f* peaks for the W-capped MTJ. This sample exhibits similar changes in XPS intensities as observed for the Ta-capped MTJ. These results suggest that maintaining the large TMR ratio of the Ta- and W-capped MTJs at high T_{ann}'s is due to reduced diffusion of the capping layers.

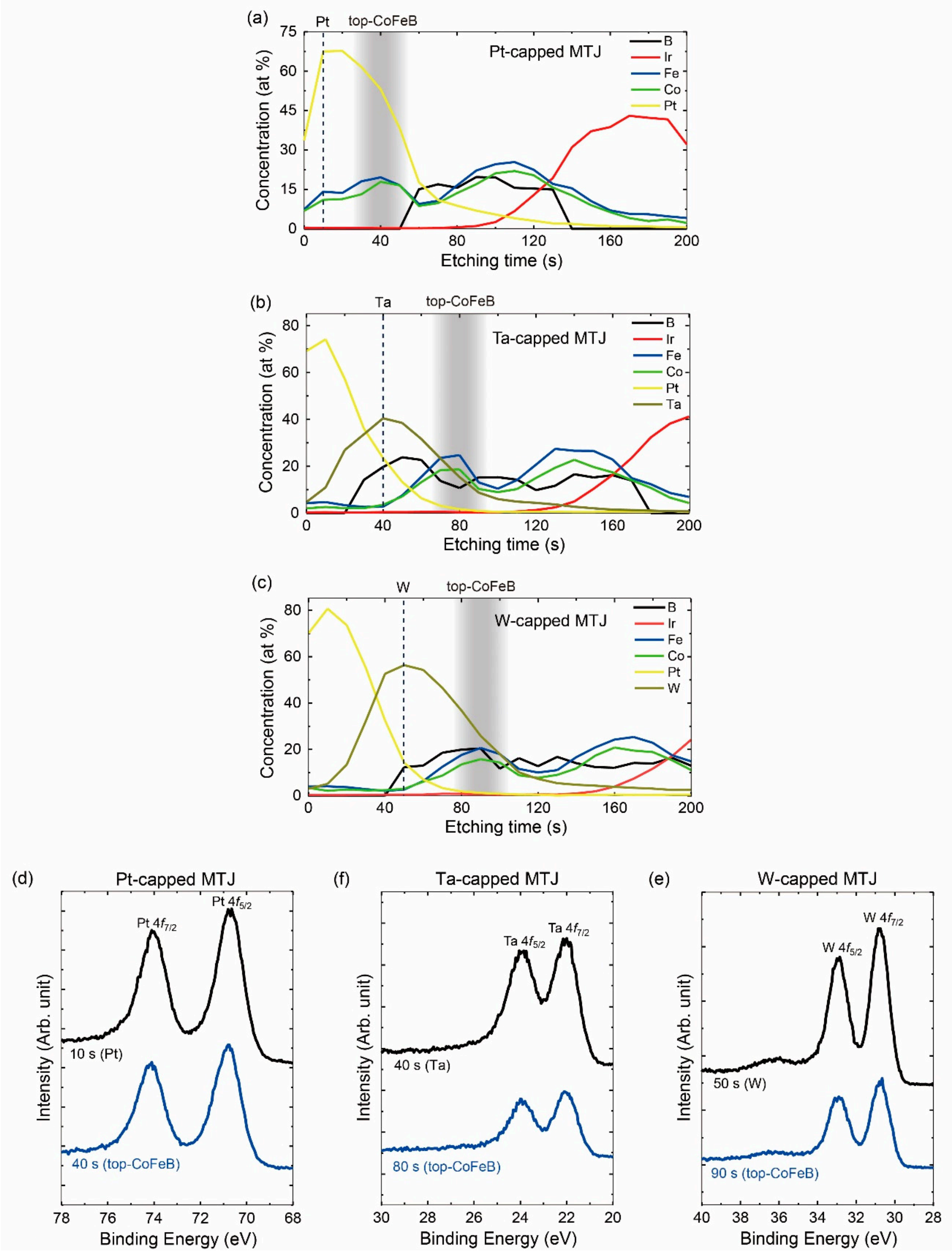

Figure S1. (a-c) The XPS depth profile of MTJ films with different capping layers of Pt (a), Ta (b), and W (c). (d-f) The XPS spectra measured in the capping and top CoFeB layers. (d) Pt 4*f* peaks of Pt-capped MTJ films, (e) Ta 4*f* peaks of Ta-capped MTJ films, and (f) W 4*f* peaks of W-capped MTJ films. All films were annealed at 450°C.

Supplementary Information S2. The diffusion of B atoms and crystallization of the CoFeB layers

In order to elucidate the distribution of B before and after annealing, we conducted XPS depth profile experiments on both as-deposited and annealed (T_{ann} of 450°C) samples. Note that we determined the position of each layer within the MTJ films as described on Supplementary Information 1. Figures S2(a,b) present the XPS spectra of B 1*s* measured in the capping and top-CoFeB layers. We observed a negligible B 1*s* peak intensity in the Pt capping both before and after annealing, indicating that the Pt capping layer cannot absorb B effectively. In contrast, for the Ta-capped MTJ film [Figure S2(c,d)], there are a significant increase (decrease) in the intensity of B 1*s* peak in the Ta (CoFeB) layer after annealing. This demonstrates that the Ta layer serves as an effective boron absorber during the annealing process, promoting the crystallization of the CoFeB layer and consequently enhancing the TMR ratio [1–3].

Next, we analyzed the microstructural changes of the CoFeB layer using bright-field STEM images of the as-deposited and annealed (T_{ann} =450°C) MTJ films. Note that the same films as those shown in Figures 2 and 3 of the original manuscript. Figures S3(a) and S3(b) present the bright field STEM images of the Pt-capped MTJs before and after annealing at 450°C, respectively. While the IrMn, bottom CoFeB, and MgO layers are clearly distinguished in both films, it is difficult to observe microstructural changes in the top CoFeB layer due to significant intermixing with the Pt capping. However, it is found that the bottom CoFeB layer was partially crystallized after annealing in Figure S3(b). On the other hand, for the Ta-capped MTJs, it is clearly observed that the microstructure of the top CoFeB layer changes from an amorphous phase [Figure S3(c)] to a crystalline bcc phase [Figure S3(d)] upon annealing. These microstructural changes according to the capping layers are consistent with the differences in the annealing temperature dependence of the TMR ratio with different capping layers shown in Figures 1b and 3c.

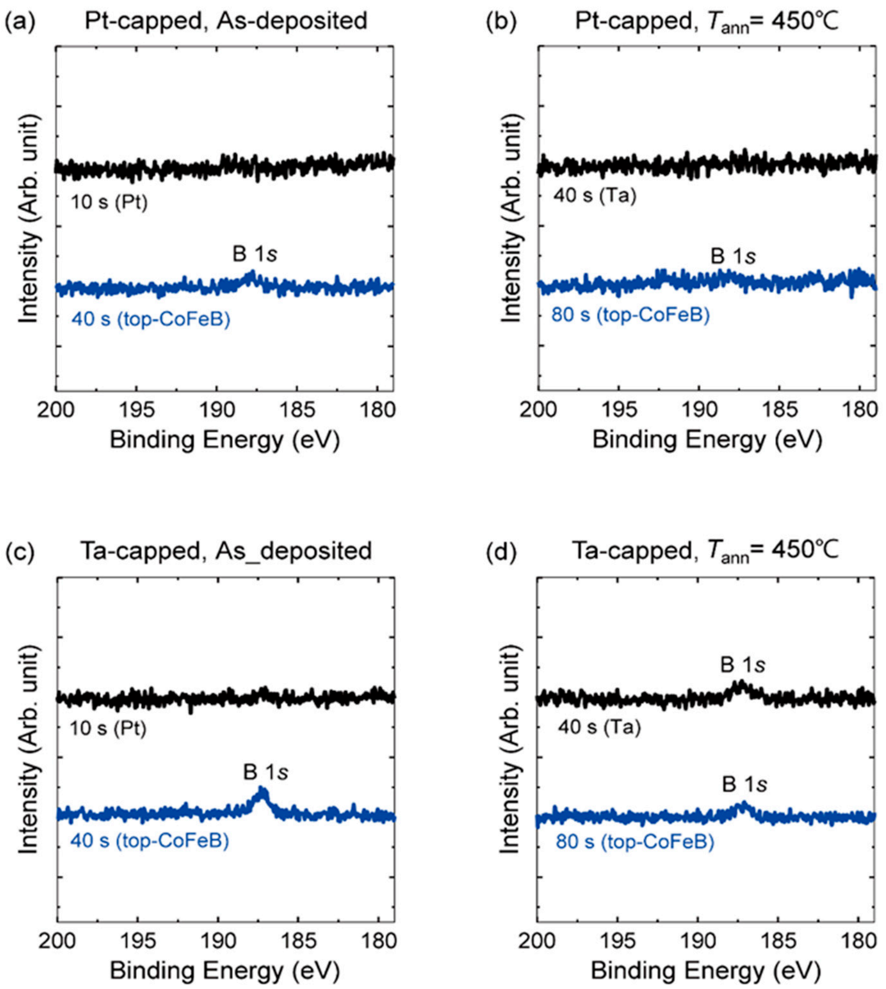

Figure S2. The B 1*s* XPS spectra measured in the capping and top CoFeB layers. (a, b) Pt-capped MTJ before (a) and after (b) annealing, (c, d) Ta-capped MTJ before (c) and after (d) annealing. T_{ann} = 450 °C.

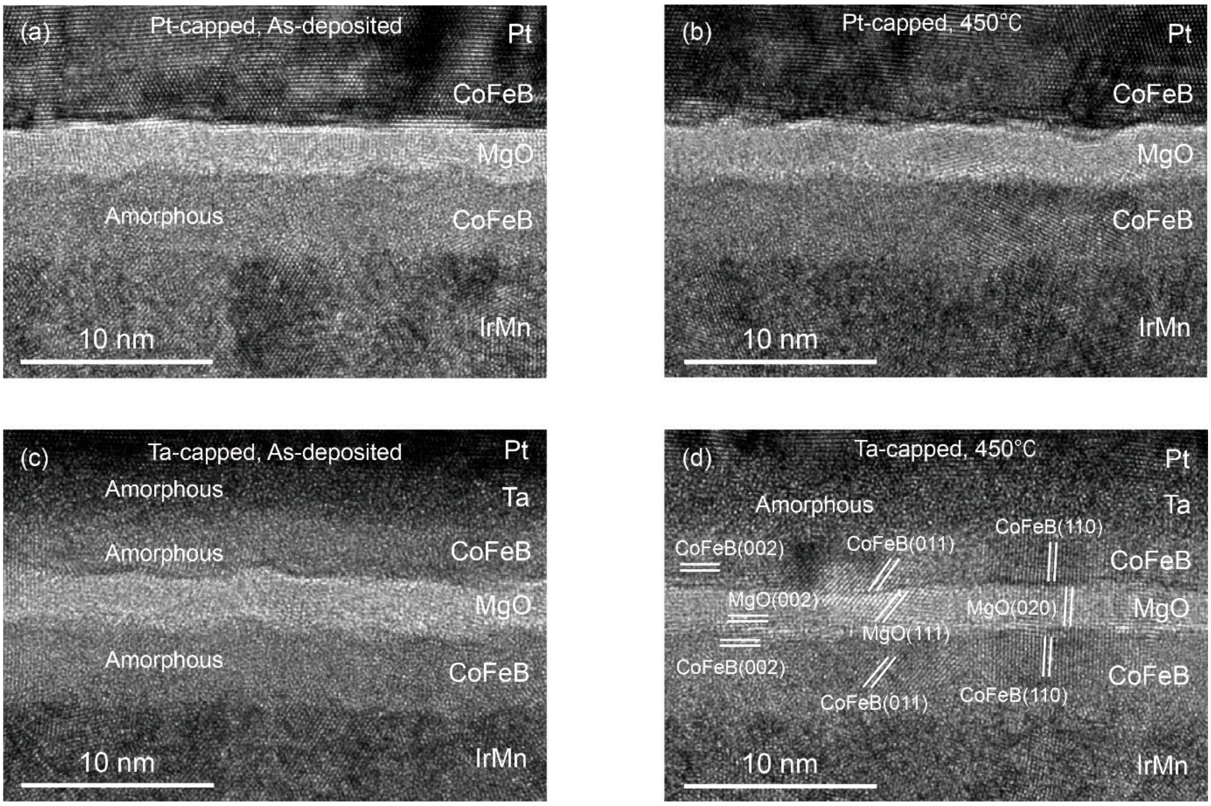

Figure S3. The cross-sectional bright-field scanning transmission electron microscope images of (a) as-deposited and (b) annealed Pt-capped MTJ films, and (c) as-deposited and (d) annealed Ta-capped MTJ films.

Supplementary References

[1] S. Ikeda; J. Hayakawa; Y. Ashizawa; Y.M Lee; K. Miura; H. Hasegawa; M. Tsunoda; F. Matsukura; H. Ohno. Tunnel magnetoresistance of 604% at 300K by suppression of Ta diffusion in CoFeB/MgO/CoFeB pseudo-spin-valves annealed at high temperature. *Appl. Phys. Lett.* **2008**, *93*, 082508. DOI: 10.1063/1.2976435

[2] T. Miyajima; T. Ibusuki; S. Umehara; M. Sato; S. Eguchi; M. Tsukada; Y. Kataoka. Transmission electron microscopy study on the crystallization and boron distribution of CoFeB/MgO/CoFeB magnetic tunnel junctions with various capping layers. *Appl. Phys. Lett.* **2009**, *94*, 122501. DOI: 10.1063/1.3106624

[3] H. Bouchikhaoui; P. Stender; D. Akemeier; D. Baither; K. Hono; A. Hutten; G. Schmitz. On the role of Ta cap in the recrystallization process of CoFeB layers. *Appl. Phys. Lett.* **2013**, *103*, 142412. DOI: 10.1063/1.4824033
